# Spectral Unmixing to Reduce Refraction Effects in Feulgen-Stained Slides

**DOI:** 10.3390/s26010057

**Published:** 2025-12-21

**Authors:** Kouther Noureddine, Paul Gallagher, Anita Carraro, Jagoda Korbelik, Branko Palcic, Martial Guillaud, Calum MacAulay

**Affiliations:** 1 Department of Integrative Oncology, BC Cancer Research Institute, Vancouver, BC V5Z 1L3, Canada; pgallagher@bccrc.ca (P.G.); acarraro@bccrc.ca (A.C.); jkorbeli@bccrc.ca (J.K.); bpalcic@bccrc.ca (B.P.); mguillau@bccrc.ca (M.G.); cmacaula@bccrc.ca (C.M.); 2 Department of Pathology and Laboratory Medicine, Faculty of Medicine, University of British Columbia, Vancouver, BC V6T 1Z4, Canada

**Keywords:** ploidy analysis, spectral unmixing, quantitative image cytometry

## Abstract

Introduction: As DNA image cytometry and DNA image histology serve as valuable tools in clinical tumor pathology, the need for precise and accurate DNA amount measurements is crucial. This study describes the process of employing spectral unmixing on Thionin-stained slides as a means of reducing refraction effects introduced in the image, during imaging, due to changes in the refraction index within the tissue being imaged. Methods: A correction method that reduces refraction effects on the DNA quantitation measurements by making use of the spectrally limited absorption properties exhibited by Thionin relative to the more spectrally uniform effects of tissue refraction as a function of wavelength. Results: Spectral unmixing enables an improved estimate of DNA amount at every pixel and a potentially truer representation of the actual distribution of the DNA within individual cell nuclei. Conclusions: Spectral unmixing is a valuable computational technique widely used in histology and cytology research. By reducing refraction-based optical artifacts in the image, it enhances the accuracy of DNA quantitation, minimizes variability, and improves the discriminating ability of nuclear DNA organization as quantified by texture features.

## 1. Introduction

Cancer arises from alterations in the inheritable molecular characteristics of normal cells [1]. The majority of these inheritable characteristics are contained within the DNA of the cells. Changes in the structure and organization of the DNA within the nucleus can result in unregulated growth of these cells and hence cancer. Quantitative DNA analysis measures the DNA content and its spatial organization within individual cell nuclei, revealing various cellular characteristics such as ploidy status and cell cycle phase, which are identified by their fractional DNA content [2]. As cells transform from a normal to a malignant state, several alterations occur in the DNA and evaluating the total DNA content in a cell population can inform us about the extent of genetic damage the cells have undergone. Flow cytometry and image cytometry are widely used methods that allow us to quantify the DNA content within individual cells in large cell populations accurately, making it an increasingly important tool in clinical tumor analysis [3]. These methods rely on the use of dyes or stains which bind stoichiometrically to DNA, providing an accurate measurement of the DNA content within the cell [4].

The Feulgen reaction remains one of the most commonly used DNA stains in image cytometry to specifically detect and quantify DNA in a consistent, reproducible, and standardized manner [5,6]. It enables the specific staining of DNA by utilizing Schiff reaction or Schiff-like reagents such that the amount of dye found in the cell is proportional to DNA concentration [7]. While Thionin-stained absorption image cytometry is a valuable tool for DNA quantitation, it is often subject to methodological limitations such as staining variability and optical artifacts including refraction and glare effects.

Biological tissue samples exhibit non-uniform absorption and are inherently heterogeneous in the refractive index due to structural protein variations between nuclei, cytoplasm, connective tissue, and the embedding or mounting medium [8]. As light passes through biological tissue, it undergoes multiple interactions, including refraction, partial reflection, and scattering, causing local variations in the transmitted intensity that are unrelated to chromogen absorption [9]. The Beer–Lambert law describes the exponential attenuation of light as it passes through a homogeneous, non-scattering medium, assuming that all losses in transmitted intensity are due solely to absorption [10]. Under these ideal conditions, the optical density (OD) is linearly proportional to the concentration of the absorbing species and the optical path length. However, in biological tissues and cytological samples, glare, scatter, and refraction introduce additional optical contributions which violate the assumptions of the Beer–Lambert law [10]. In return, these effects results in an underestimation or overestimation of the optical density at each measurement point (pixel) and, consequently, the apparent DNA content. Although attenuation in biological tissue can be represented using a modified Beer–Lambert law to account for both absorption and scattering, our approach targets refraction-related artifacts, which represent the primary source of error in these images.

Given the importance of generating accurate measurements for precise DNA quantitation, correction methods that focus on reducing optical limitations or systematic errors are of great interest [11]. Previous studies have shown that glare and refraction within the microscope optics can significantly distort optical density measurements in stained nuclei, leading to an altered measurement of DNA content and increased measurement variability. Haroske et al., for example, demonstrated that subtracting the glare contribution from transmitted light intensity improves both the precision and accuracy of DNA cytometry measurements [12].

**Figure 1:**
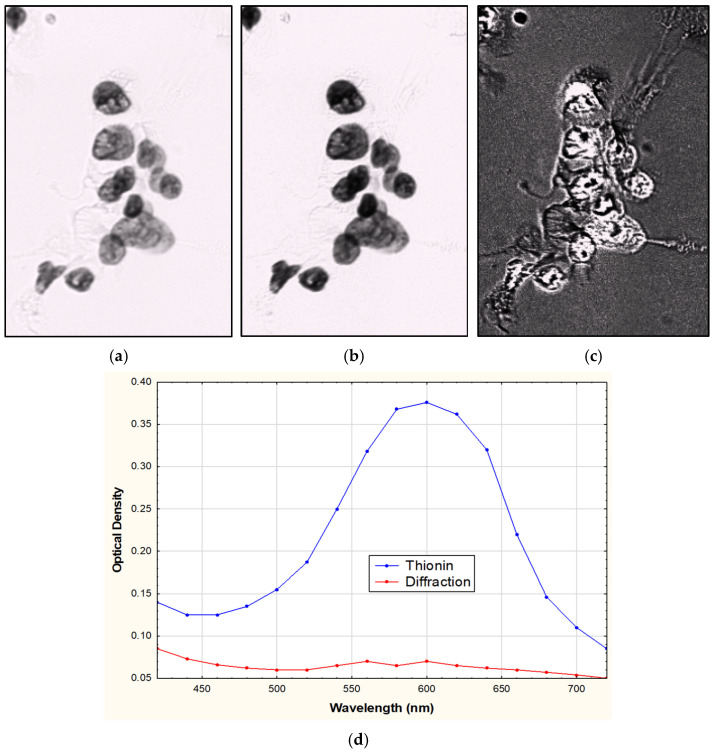
(**a**) 600 nm image, (**b**) unmixed Thionin image, (**c**) contrast-enhanced unmixed refraction effects image and (**d**) Observed optical density spectra of Thionin and refraction areas of the hyperspectral image stack.

Spectral unmixing refers to the process of computationally unmixing stains to determine the concentration of the stain at every pixel in a selected area using the absorption spectra of each stain in the image. Thionin exhibits a distinctive and sharply peaked (600 nm) absorption spectra when compared to other non-specific absorbers or refraction effects observed within pathology slides (Figure 1). Refraction effects generally do not cause light to be absorbed but cause light to not take a straight path through the sectioned tissue or cells. It causes more light to be detected at some pixels and less light at other pixels, so it has the effect of an absorber with both positive and negative concentrations. In this context negative concentration indicates more light appears to come from a pixel than it was illuminated with as light from neighboring pixels has been diverted by refraction into the measured pixel. By mathematically decomposing the measured spectrally overlapping signals in an image into their constituent absorptive and refractive components, spectral unmixing enhances the accuracy of stain quantification and enables more precise estimation of nuclear DNA content in cytology and histology slides. In this paper, we describe the process of utilizing spectral unmixing to improve the measurement of DNA content and quantification of the distribution of DNA within the nuclei of cells by correcting for refraction.

## 2. Materials and Methods

### 2.1. Cell Culture

Cells were grown on cytology slides to optimize staining protocols. HL-60 acute promyelocytic leukemia cells were obtained from the American Type Culture Collection (Manassas, VA, USA) and maintained in Iscove’s Modified Dulbecco’s Medium supplemented with 10% fetal bovine serum and 1% penicillin/streptomycin. Cultures were incubated at 37 °C in an atmosphere of 95% air and 5% CO_2_.

For slide preparation, autoclaved, uncharged, pre-cleaned glass slides were placed into square culture dishes (three slides per dish) and covered with 15 mL of cell suspension at 5 × 10^5^ cells/mL in growth medium. Phorbol 12-myristate 13-acetate (PMA; 15 µL of a 1 mg/mL solution in ethanol; Sigma-Aldrich, Oakville, ON, Canada) was added to each dish to promote cell adherence. After 48 h, slides were rinsed, fixed in Sed-Fix^®^ (Surgipath, Richmond, IL, USA) for 40 min, and air-dried overnight. Prior to staining, dried fixative was removed by immersing slides in ethanol for 20 min at room temperature followed by air-drying.

HL-60 slides were used because they provide a convenient and reproducible material and were routinely employed in our laboratory to monitor batch-to-batch variation in Thionin staining.

### 2.2. Thionin Staining

Thionin acetate (Sigma-Aldrich) was used to formulate the staining reagents. All solutions were prepared one day in advance. For the preparation of approximately 250 mL of Thionin stain, 0.125 g of Thionin powder was dissolved in 110 mL of deionized water and heated to boiling for 5 min. After cooling to room temperature, 32.5 mL of 1 N hydrochloric acid, 110 mL of tert-butanol, and 2.175 g of sodium bisulphite were added. The mixture was stirred for one hour, kept overnight, and filtered immediately before staining.

All staining procedures were carried out at 23–24 °C using a temperature-controlled water bath, with extensive rinses in deionized water between steps. Slides were post-fixed for one hour in Böhm-Sprenger fixative (methanol, formalin, and acetic acid at a 16:3:1 ratio), hydrolyzed in 5 N hydrochloric acid for one hour, and then stained in the Thionin solution for one hour. This was followed by three rinses in bisulphite solution (0.5% sodium bisulphite in 0.05 N hydrochloric acid), each separated by water washes. After a final wash, slides were dehydrated through three 30-s ethanol baths, cleared in xylene, and cover slipped for imaging.

### 2.3. Hyperspectral Imaging and Analysis

Hyperspectral imaging was performed using our in-house developed automated hyperspectral whole-slide imaging system. The hyperspectral scanner consists of a brightfield microscope (Zeiss Axioscope 2 Mot plus microscope and Zeiss 20X (NA 0.75) (Carl Zeiss Microscopy, Jena, Germany) plan APROCHROMAT objective lens), a CRI Varispec tunable liquid crystal filter (tunable between 420 nm and 720 nm in 20 nm increments), and an Andor Neo camera with a computer-controlled x-y stage (Marzhauser Wetzlar SCAN series ((Märzhäuser Wetzlar GmbH & Co. KG, Wetzlar, Germany)). Images were acquired with a pixel sampling spacing of 0.233 μm (20× magnification) in both x and y coordinates. The Region of Interest (ROI) to be imaged is selected by the user prior to imaging. The system automatically collected 16 different spectral images from 420 to 720 nm per camera field and covered the defined area to be imaged with sequentially acquired overlapping camera fields. Once the ROI was imaged, the system software aligned and stitched all overlapping camera fields together to make a seamless hyperspectral image. A spectral image stack from an empty area of each of the slides was also collected for flatfield calibration for each wavelength specific image (images with the light source blocked).

As the Thionin stain is stoichiometric for DNA, DNA content is proportional to the integrated optical density (IOD) of the cell. Using the spectral flatfield calibration images, we calculated the optical density for every pixel for each wavelength in the images covering the selected ROI. These optical density images were the inputs to the spectral unmixing process.

### 2.4. Spectral Unmixing

Spectral unmixing is performed to separate objects in the sample based on their spectra using the formula:

(1) D=C×S+E

*D* is the Optical density Image data (M × N), *C* is the concentration matrix (M × K), *S* is the spectra Matrix (K × N) and *E* is the Noise (M × N) (K components, N wavelengths and M image size in pixels) [13,14].

The optical density image data is reconstructed from the concentration data and spectral matrix; in our case, we used a spectral matrix with two components (k = 2).

Using the observed absorption spectra of Thionin (measured by averaging over multiple pixel areas occupied by nuclei) and the observed loss of light intensity spectra from areas of unstained cell cytoplasm (which we have assumed is due to refraction caused by the difference in index of refraction of cell cytoplasm and the surrounding paramount used to affix the coverslip) the stains are computationally unmixed to determine the concentration of stain for every pixel in the selected area and the amount of refraction occurring at each pixel location. Although Feulgen staining employs a single chromogen/dye (Thionin, absorption peaked at 600 nm), the measured spectrum is not composed of a single component. Biological tissues and cytological samples exhibit close to wavelength-independent refraction that add optical contributions to the apparent peaked absorbance signal of Thionin. As a result, measuring the spectrum at each pixel represents a combination of the true Thionin absorption spectra and an additional refraction-related component. Spectral unmixing is therefore capable of separating these components, with the Thionin absorbance spectrum treated as one spectrally distinct component and the refraction contribution treated as a second spectrally dispersed component.

The spectral unmixing assumes that every pixel in the flatfield- and dark field-corrected recorded images (16 wavelengths) was a linear combination of the concentration of the individual stains occurring at that pixel weighted by the absorption characteristics of each of the stains occurring at that pixel. When mathematically separating the components, it adds an additional error term to each pixel to compensate for the electronic and photonic noise in the images. The concentration of the Thionin was required to be non-negative while the concentration of the refraction effects was allowed to be both positive and negative.

The Multivariate Curve Resolution–Alternating Least Squares algorithm (MATLAB R2014a) was used to separate the linear combinations of absorption stains, each varying in concentration at individual pixels. The analysis involved transforming the collected hyperspectral image data using a logarithm base 10 scale to make the result linear with amount of absorption occurring at each pixel (Beer–Lambert law is commonly applied to chemical analysis measurements to determine the concentration of chemical species that absorb light) [15,16]. This data was then modeled to represent concentration images for each stain, each multiplied by the corresponding stain spectra plus an error term [13,14].

The spectral component used to model refraction in the unmixing procedure was derived empirically from regions of cytoplasm that should be devoid of Thionin. These areas exhibited a weak wavelength-dependent signal, which we attribute to light redistribution caused by local refractive index variations within and between the cytoplasm and the surrounding medium. This component is not intended to represent a universal physical spectrum of refraction, but rather a residual term that captures the dominant optical contributions present in the image. The purpose of including this term is to remove refraction-induced distortions from the Thionin channel and thereby improve nuclear contrast (potentially making segmentation easier) and measurement consistency.

## 3. Results

### 3.1. Spectral Unmixing Improves Nuclear Contrast and Appears to Unmix Refraction Effects

In Figure 1, a cytology slide was stained with Thionin and imaged at 16 different wavelengths from 420 nm to 720 nm. Using the hyperspectral image, the spectra for Thionin was generated by selecting pixels that correspond to areas within the nuclei. Similarly, by selecting areas which appear to exhibit refraction (areas of cytoplasm which should not have any Thionin stain present), we were able to generate the spectra of what we are referring to as refraction. These regions, which should ideally be unstained, displayed spectral features characteristic of wavelength-dependent light redistribution caused by refractive index mismatches between the cytoplasm and the surrounding medium. During spectral unmixing, the measured pixel spectra were modeled as linear combinations of these two components—Thionin absorption and refraction. While concentration estimates for chromogenic stains are typically constrained to positive values, in this case, the refraction component was allowed to assume negative coefficients. This adjustment accounts for regions exhibiting apparent intensity values exceeding the incident illumination, corresponding to areas where refracted or diffracted light was redirected into the optical path, producing additive signal contributions in the brightfield image.

After acquiring the multispectral images and performing spectral unmixing, the unmixed Thionin image revealed enhanced contrast between the nuclei and background. Additionally, the cytoplasmic refraction effects are reduced in the image even though the nuclear contrast is much higher (Figure 1b). Notably, this improvement could not be achieved simply by increasing contrast in the original image, confirming that spectral unmixing isolates the true absorptive signal from refractive artifacts.

Additionally, if we look at what is being attributed to the refraction effects/withdrawn from the image (Figure 1c), it resembles what we would expect a refraction image to portray if we were using dark field or stoppered down the numerical aperture of the microscope greatly, further supporting the interpretation that this spectral component captures refractive light redistribution. Together, these findings suggest that spectral unmixing effectively suppresses refraction-induced distortions while improving nuclear contrast.

**Figure 2:**
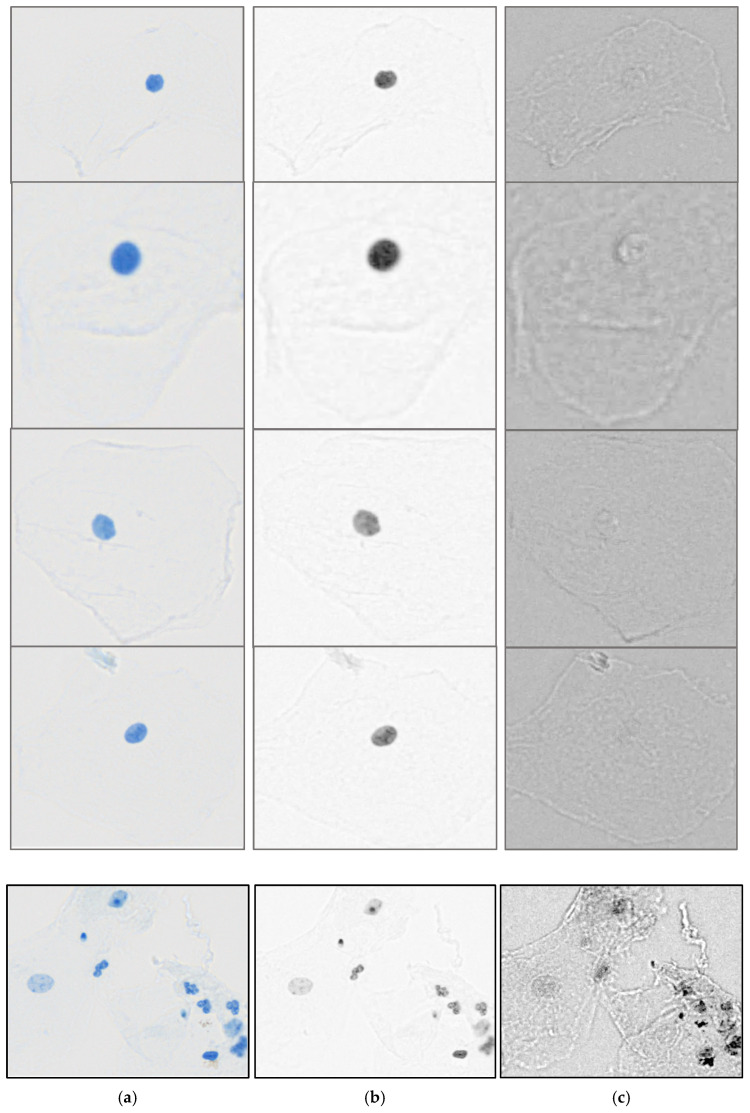
(**a**) RGB Color Image from Zeiss Axioscan 7, (**b**) Unmixed Thionin Concentration Image and (**c**) Unmixed Other (Refraction Effects) Image.

To further evaluate whether our method yields comparable results when applied to standard three-channel RGB images, cytology samples were also imaged using a ZEISS Axioscan 7 (Carl Zeiss Microscopy, Jena, Germany)) brightfield slide scanner. As shown in Figure 2, spectral unmixing performed on the RGB image yielded an unmixed Thionin channel with strong contrast between nuclei and background, comparable to that obtained from the hyperspectral dataset. The corresponding refraction component exhibited a characteristic halo-like appearance, similar to what would be expected from refracted light in brightfield microscopy. These results demonstrate that the proposed approach can be applied not only to multispectral data but also to standard RGB imagery, effectively separating absorptive (Thionin) and refractive contributions to improve nuclear contrast and image interpretability.

**Figure 3:**
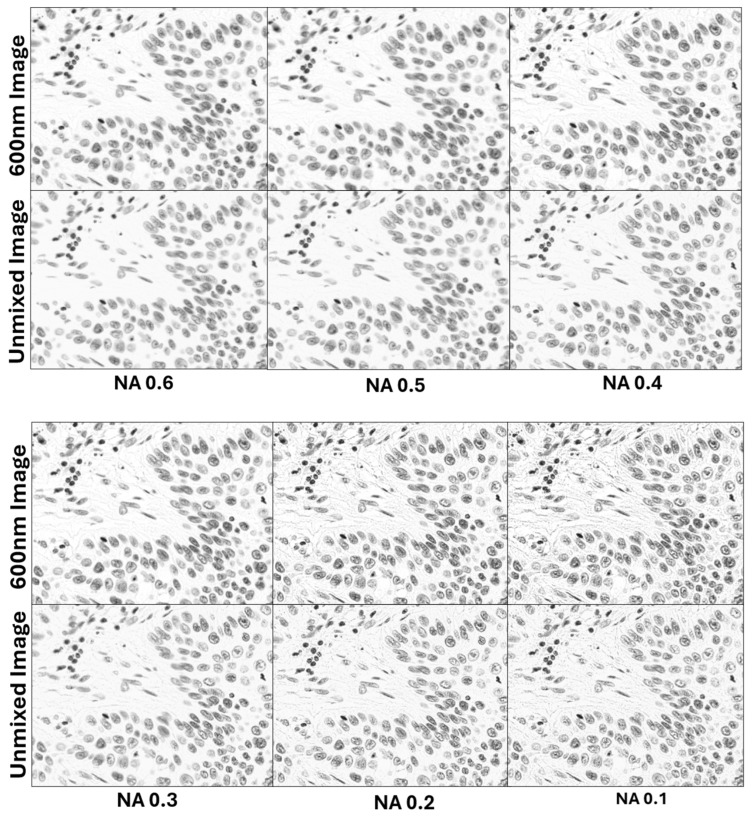
Reduction in refraction artifacts by spectral unmixing across numerical apertures. Representative 600 nm and unmixed images of Thionin-stained nuclei acquired at different NAs (0.6–0.1). Refraction and glare effects increase as NA decreases in the 600 nm images but are substantially reduced after spectral unmixing, resulting in improved nuclear contrast and clearer morphology.

To further evaluate the effectiveness of spectral unmixing in reducing refraction-related artifacts, the same field of view of a Thionin-stained lung tissue section was imaged under six different illumination conditions, each with progressively decreasing condenser numerical aperture (NA). In the first condition, the NA was set to 0.6, closely matching that of the objective lens, and then decreased in increments of 0.1 for each subsequent acquisition, with the final condition recorded at a very low NA of 0.1. As shown in Figure 3, decreasing the NA resulted in a progressive increase in the visibility of refractive features in the raw 600 nm images, manifesting as enhanced edge brightening and glare around cellular structures. Following spectral unmixing, however, these refractive artifacts were markedly diminished, while nuclear contrast remained high. This observation provides further evidence that the unmixing process effectively separates Thionin and refractive contributions, substantially reducing artifacts associated with refraction.

**Figure 4:**
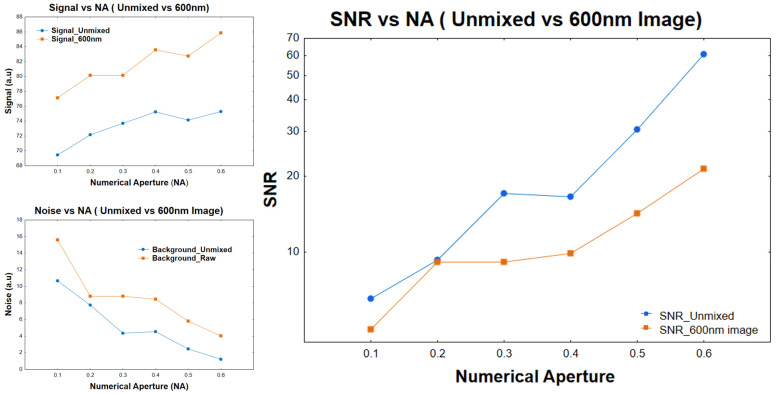
Effect of numerical aperture on signal, noise, and signal-to-noise ratio (SNR) before and after spectral unmixing. Signal, noise, and SNR were measured from nuclei and background regions in Thionin-stained cytology slides imaged at varying numerical apertures (NA). While the unmixed images showed slightly lower signal intensity, background noise was markedly reduced, resulting in consistently higher SNR values compared to the 600 nm images. The improvement was most pronounced at higher NAs, confirming that spectral unmixing enhances image quality by reducing refractive and illumination-induced noise.

To quantitatively assess the improvement in image quality achieved through spectral unmixing, the signal-to-noise ratio (SNR) was calculated for both the original and unmixed images across all NA conditions. For each image, the signal was defined as the mean pixel intensity within nuclear regions, while noise was estimated as the standard deviation of background regions devoid of staining. As shown in Figure 4, the SNR of the unmixed Thionin images was consistently higher than that of the corresponding 600 nm images at every NA tested, with it being more pronounced at higher NA’s. The improvement in SNR reflects the ability of the unmixing process to suppress non-absorptive intensity fluctuations introduced by refraction, thereby isolating the true absorptive component of the signal and minimizing the fluctuations observed in the non-Thionin-stained areas. The improvement in the SNR primarily comes from a reduction in the noise term.

### 3.2. Spectral Unmixing Enhances DNA Content Measurements

**Figure 5:**
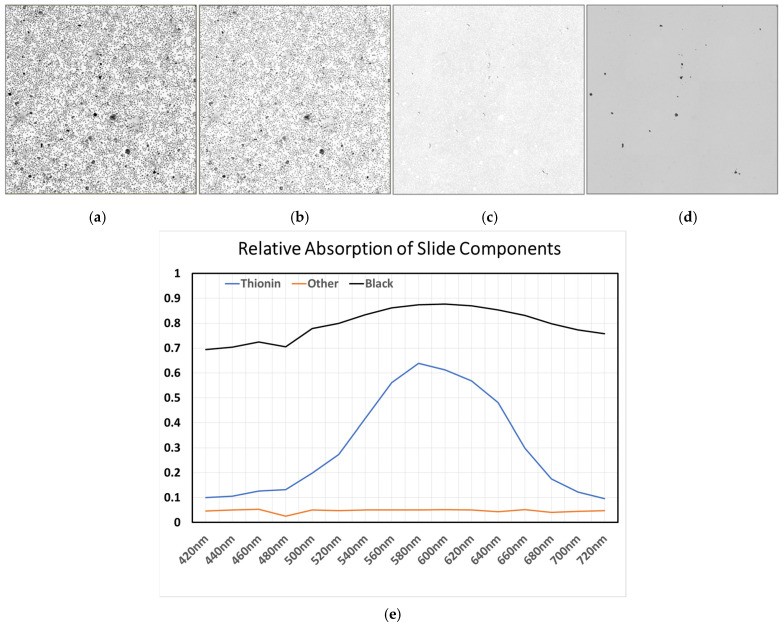
HL60 cells stained with Thionin and unmixed: (**a**) 16 averaged 600 nm images (**b**) unmixed Thionin concentration image (**c**) unmixed refraction concentration image, (**d**) unmixed black concentration image and (**e**) relative absorption of the observed components (blue—Thionin stain absorption, orange—absorption observed for unstained cytoplasm, and black—absorption of dark particles/debris).

One potential concern when performing spectral unmixing is the loss of any data measurement fidelity as part of the unmixing process. To determine whether removal of the refraction-related effects during the unmixing process preserves the DNA content measurements during the quantitation process, HL60 cells grown to confluence on microscope slides were stained with Thionin and imaged using a 16-wavelength hyperspectral acquisition (Figure 5). After acquiring the spectra of three components in the 16-wavelength hyperspectral image (Figure 5a) which we designate here as “Thionin”, “black”, and “other”, spectral unmixing is performed to separate all the black debris and possible refraction effects from the Thionin concentration data. In the resulting unmixed Thionin image, nuclear regions were clearly delineated, while the “other” component image captured non-chromatin contributions such as debris and refractive scatter. Closer inspection of the unmixed “other” concentration image (Figure 5c) reveals intensity variations surrounding nuclear boundaries, which may indicate that refractive contributions were reduced in these regions during unmixing. Such changes could arise from the reduction in refraction effects in this part of the image due to changes in index of refraction caused by differing densities of DNA molecules in different parts of the nucleus, potentially altering DNA distribution measurements made on the nuclei images.

These adjustments primarily represent correction of optical distortions rather than modification of the true absorptive signal. Although minor intensity redistribution occurs near nuclear peripheries, the overall DNA content quantitation derived from the unmixed Thionin channel appears consistent with Beer–Lambert absorption behavior, suggesting that the unmixing process preserves measurement fidelity while effectively mitigating refractive artifacts.

To quantify the potential the effects of spectral unmixing on DNA measurements, four independent Thionin-stained cytology slides were imaged and analyzed. For each slide, a 16-wavelength hyperspectral image stack and a single-wavelength 600 nm image. As one could argue that the unmixed Thionin image is a weighted sum across 16 images and potentially has a superior signal-to-noise ratio because of this weighted averaging, 16 600 nm images were acquired and averaged to make the single-wavelength 600 nm image as equivalent as possible. Thus, the averaged 600 nm image signal-to-noise ratio (SNR) would be comparable to or greater than that of the unmixed image, which draws information from all 16 spectral channels.

**Figure 6:**
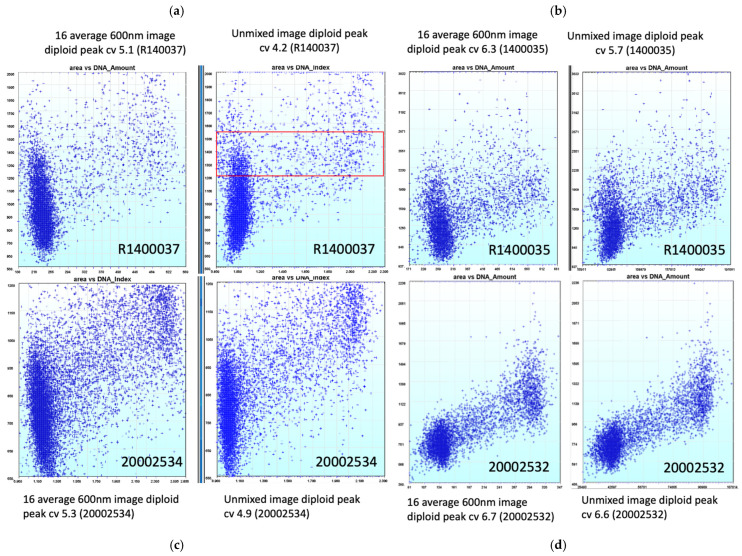
(**a**) Plot of area of nuclei vs. DNA amount for slide R1400037 in the 16 averaged 600 nm image vs. spectrally unmixed image. (**b**) Plot of area of nuclei vs. DNA amount for slide R1400035 in the 16 averaged 600 nm image vs. spectrally unmixed image. (**c**) Plot of area of nuclei vs. DNA amount for slide 20002534 in the 16 averaged 600 nm image vs. spectrally unmixed image. (**d**) Plot of area of nuclei vs. DNA amount for slide 20002532 in the 16 averaged 600 nm image vs. spectrally unmixed image. The red box represents the approximate area, DNA amount range selected to compare features measures on diploid vs. tetraploid nuclei.

For all these images, nuclei were segmented and ~100 features were calculated for each nucleus [17]. For each slide, total DNA content was plotted as a function of nucleus area (Figure 6). In slide R140037, analysis of the averaged 600 nm image revealed that the diploid cell distribution was tilted, indicating a dependency of the measured DNA amount on nuclear size—larger nuclei appeared to contain less DNA and the whole diploid distribution has a coefficient of variation (cv) of 5.1. However, after spectral unmixing, the diploid peak appears much more vertical in its alignment and its cv is tighter at 4.2 (Figure 6a). Similar trends were observed across all four slides (Figure 6b–d), suggesting the unmixing tends to reduce the nucleus size effects on the DNA measurements of the diploid or tetraploid peaks while decreasing their CVs. Thus, spectral unmixing appears to improve DNA measurements.

### 3.3. Spectral Unmixing Improves Ploidy Analysis

For DNA ploidy analysis, the coefficient of variation (CV) improved across all slides following spectral unmixing (Table 1). Furthermore, when comparing the ratio of the diploid to tetraploid peaks between the 16-image-averaged 600 nm image and the unmixed image, the unmixed results were consistently closer to the theoretical value of 2, as expected for accurate DNA quantitation. Smaller CVs and a ratio closer to 2 indicate more precise measurements as the amount of DNA for each nuclei in the diploid peak is essentially the same, down to the base pair level, and just prior to division, the amount of DNA is twice that of the diploid nuclei, again, down to essentially the base pair level. This finding indicates that the removal of refraction-related artifacts yields measurements that more accurately reflect true DNA content. To assess the statistical significance of these improvements, both pairwise *t*-tests (*p* = 0.036) and Willcoxon matched pair test (*p* = 0.012) were performed, each confirming that the observed differences between the raw and unmixed images were statistically significant. Together, these results demonstrate that spectral unmixing enhances the quantitative fidelity of Thionin DNA ploidy measurements by reducing optical distortions and improving measurement precision.

### 3.4. Spectral Unmixing Enhances Texture Features

**Figure 7:**
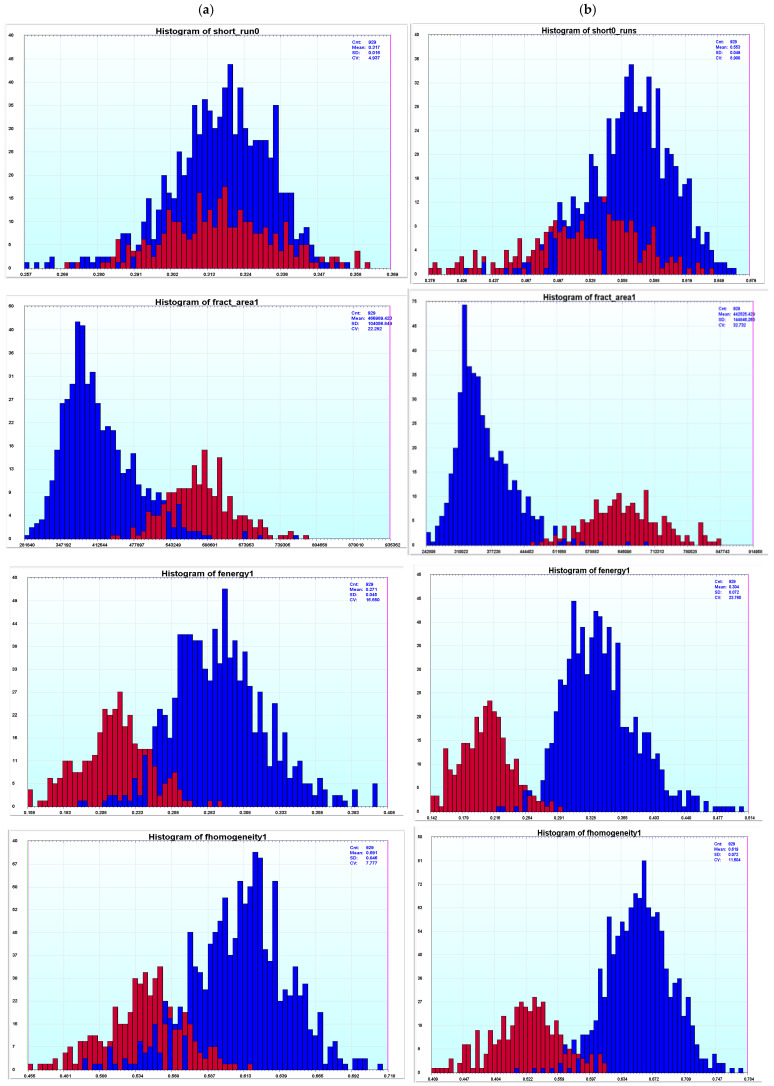
Texture features for slides that have increased separation after unmixing for slide 1400037. (**a**) Texture features from 16 averaged 600 nm images image data for slide 1400037. (**b**) Texture features of spectrally unmixed image data for slide 1400037 (nuclei features from diploid peak nuclei are depicted in blue and nuclei features from tetraploid peaks nuclei are shown in red).

To examine the effect of spectral unmixing on texture features which quantify the DNA distribution within the nuclei, we compared the extracted texture features for cell nuclei of the same general size range (see the red box in Figure 6a) corresponding to the diploid or tetraploid distributions. As many of the features have a potential correlation with the size of the nucleus, we tried to limit the area range of the examined nuclei (Red Box in Figure 6a) while maintaining a reasonable number of both diploid and tetraploid nuclei for analysis. For each slide, texture features were computed from both the 16-image-averaged 600 nm dataset and the spectrally unmixed dataset. As shown in Figure 7, we can see the separation between the diploid and tetraploid data (nuclei from diploid peak in blue and nuclei from tetraploid peak in red) for both the 600 nm image data and the spectrally unmixed data. Although we can distinguish the nuclei from the diploid and tetraploid distributions in the 600 nm data for some of the features, in the spectrally unmixed data, the separation improves for most of the texture features and many of the texture features become more discriminating (Figure 7). These results indicate that spectral unmixing not only improves signal consistency for DNA quantitation but also enhances the sensitivity of texture-based metrics, likely due to a reduction in the DNA measurement noise from the refraction-induced light redirection influencing pixel DNA measurements within nuclear chromatin patterns.

**Figure 8:**
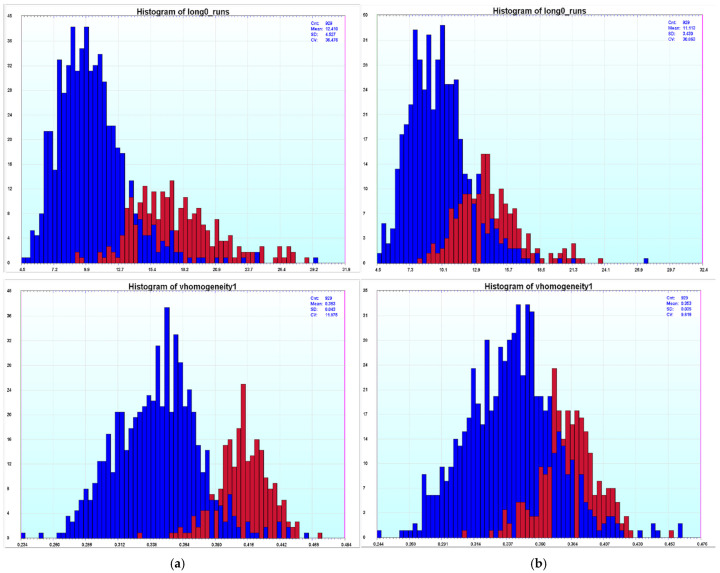
Texture features for slides that have worse separation after unmixing for slide 1400037: (**a**) average of 16 600 nm image data and (**b**) spectrally unmixed image data1400037 (nuclei features from diploid peak nuclei are depicted in blue and nuclei features from tetraploid peaks nuclei are shown in red).

Although for the majority of the texture features the differentiating ability improves with unmixing, there are a few where it becomes poorer. In Figure 8, we can see that we do have texture features that, after unmixing, the separation between the nuclei from the diploid peak and the tetraploid peak becomes worse. However, in general for the texture features, across the four slides, more of the texture features improve than become worse; the improvement is larger for some of the slides than the others, but there is always more improvement than deterioration for each slide. Thus, spectral unmixing seems to be enhancing texture features.

## 4. Discussion

Here, we describe the utility of spectral unmixing in reducing refraction-related artifacts and improving the accuracy of DNA content measurements and potentially texture feature discrimination ability. Data derived from spectrally unmixed images provided more consistent and reliable estimates of DNA content, offering a representation that more closely reflects the true spatial distribution of DNA within individual cell nuclei. Although Thionin-stained absorption image cytometry remains a well-established and valuable tool for DNA quantitation, it is often limited by procedural limitation such as staining variability, illumination non-uniformity, and calibration inconsistencies. A common highlighted issue is that the measurements are affected by glare and refraction effects in the microscope system. By explicitly modeling and computationally reducing the contributions of refraction effects through spectral unmixing, we achieved a marked improvement in measurement accuracy and image interpretability.

From a DNA ploidy analysis perspective, substantial effort has been devoted to mitigating the effects of glare and refraction artifacts that compromise the accuracy of quantitative cytometry [11,12,18]. Glare, caused by unwanted light scattering and reflection within the optical system, can distort the intensity measurements of DNA-stained cells, leading to inaccuracies in ploidy assessment [19]. Refraction, caused by variations in the index of refraction of the cell cytoplasm and nuclei DNA and surrounding medium, can redirect or focus light within the sample, thereby degrading measurement accuracy. Previous methods have proposed correction methods for optical glare which have been applied and tested for DNA cytometry. These procedures rely on subtraction of the mean glare transmittance from each object to eliminate errors due to different nuclear Thionin-staining intensities [18]. Other correction methods have focused on reducing glare and refraction effects depending on size and mean optical density [12].

Spectral unmixing leverages the distinct spectral profiles of refraction which appear to exhibit a more uniform spectra appearance when compared to Thionin, which displays a characteristic absorption peak near 600 nm. This approach assumes a linear relationship between pixel intensities and the spectral contributions of each stain while compensating for noise introduced by factors such as refraction and effects usually attributed to glare. By separating the pure spectra of each component in the image, spectral unmixing reduces the optical contributions and distortions caused by refraction. This enables selective isolation of the pure Thionin absorption spectrum, thereby reducing optical distortions and improving the fidelity of DNA content measurements. Beyond ploidy analysis, texture features describing the spatial distribution of chromatin within nuclei are widely used in cytopathology to provide diagnostic and prognostic information for human cancers [20]. In addition to improving ploidy analysis, by removing the background noise and refraction effects in the image, it appears to effectively enhance texture features discriminating ability. While the results here are based on hyperspectral images taken over 16 wavelengths as a proof of concept, the same process appears to work with only three spectral bands (RGB) as seen in Figure 2. So, this approach could have broad applicability as it works with conventional color cameras for Feulgen-stained samples or any other stain with a relatively peaked absorption as a function of wavelength.

Overall, spectral unmixing has emerged as a powerful computational approach in many applications of histology and cytology research. By successfully isolating overlapping signals and removing optical artifacts introduced in the image by refraction, it significantly enhances the precision of DNA quantitation, reduces measurement variability, and appears to improve the utility of texture feature measurements. These findings suggest that incorporating spectral unmixing into standard image cytometry workflows may provide a more accurate framework for quantitative pathology. From a computational perspective, spectral unmixing is scalable and can be easily applied to larger datasets and whole-slide images. The primary concerns are image size and processing time, which can be addressed through increased memory or GPU acceleration. As such, the approach is compatible with high-throughput workflows and can be extended to larger studies without methodological limitations.

In summary, this study introduces a novel application of spectral unmixing for quantitative image cytometry, demonstrating that modeling refraction as a separate spectral component effectively corrects optical distortions inherent to brightfield imaging. By doing so, it enhances the precision of DNA ploidy quantitation and strengthens the discriminative power of nuclear texture features, both of which are critical for diagnostic and prognostic cytopathology. As spectral unmixing methods can be easily integrated and applied, incorporating this correction framework may help standardize DNA content measurements and refine image-derived metrics for cancer diagnostics and digital pathology.

## Figures and Tables

**Table 1:** Spectral Unmixing Improves DNA Ploidy Analysis: For each slide, the diploid distribution cv and the ratio of the diploid to tetraploid peaks (4c distribution mean/2c distribution mean) were calculated.

Slide	16 Frame Average 600 nm Image cv, Ratio	Unmixed Image cv, Ratio
R1400037	5.1 cv, ratio 2.06	4.2 cv, ratio 2.01
20002534	5.3 cv, ratio 2.07	4.9 cv, ratio 2.01
R1400035	6.3 cv, ratio 2.12	5.7 cv, ratio 2.02
20002532	6.7 cv, ratio 2.16	6.6 cv, ratio 2.06

## Data Availability

The data used in this study can be made available upon reasonable request.

## Abbreviations

The following abbreviations are used in this manuscript:

DNA	Deoxyribonucleic acid
OD	Optical Density
ROI	Region of Interest
IOD	Integrated Optical Density
NA	Numerical Aperture
SNR	Signal-to-Noise Ratio
CV	Coefficient of Variation
